# Disease Burden Estimation of Hepatocellular Carcinoma Attributable to Dietary Aflatoxin Exposure in Sichuan Province, China

**DOI:** 10.3390/nu16244381

**Published:** 2024-12-19

**Authors:** Mei Qin, Li Lin, Liang Wang, Yu Zhang, Lishi Zhang, Yang Song, Jinyao Chen

**Affiliations:** 1 West China School of Public Health and West China Fourth Hospital, Sichuan University, Chengdu 600041, China; qinmei9359@163.com (M.Q.); bu_liang_wang@163.com (L.W.); lishizhang_56@163.com (L.Z.); 2 Chongqing Center for Disease Control and Prevention, Chongqing 400707, China; 3 Sichuan Center for Disease Control and Prevention, Chengdu 600044, China; linli_1982@163.com (L.L.); cassea@163.com (Y.Z.)

**Keywords:** aflatoxin, dietary exposure, lifetime average daily dose, hepatocellular carcinoma, disease burden, disability-adjusted life year

## Abstract

Background: Aflatoxin B_1_ (AFB_1_), AFB_2_, AFG_1_, and AFG_2_ are Group 1 human carcinogens, with AFB_1_ notably increasing hepatocellular carcinoma (HCC) risk. Sichuan Province, China, with its subtropical monsoon climate, is susceptible to AF contamination in various food items. However, the HCC disease burden attributable to lifetime chronic dietary AF intake in Sichuan has not been investigated. Methods: The contamination data of AFB_1_, AFB_2_, AFG_1_, AFG_2_, and AFM_1_ across 20 food categories were analyzed from 2012 to 2023 in Sichuan. Along with the consumption data gathered from the 2011 China National Nutrition and Health Survey, the FDA-iRISK simulated the lifetime chronic dietary exposure patterns of ∑_5_AF and estimated the associated HCC burden using disability-adjusted life year (DALY) as the metric. Results: As for the mean AF contamination level in food from Sichuan, the estimated lifetime average daily dose (LADD) of ∑_5_AF intake was 9.77 ng/kg bw/day at minimum and 26.0 ng/kg bw/day at maximum, resulting in the lifetime HCC risks per person of 0.106% and 0.283%. The corresponding HCC burdens were 16.87 DALY/100,000 people/year and 44.95 DALY/100,000 people/year, respectively. In the same scenario, the LADD and the risk of HCC in males were higher than in females, but the PAF was higher in females. However, the high (P_95_) AF contamination level in food caused 2–3 times higher LADD and HCC burden than the mean level of AF occurrence. Among the studied food categories, grains and their products were the primary dietary sources of dietary AF exposure. Conclusions: Sichuan population’s lifetime exposure to ∑_5_AF results in an HCC burden higher than the global level. It is recommended to continuously monitor and control AF contamination in Sichuan, particularly those highly vulnerable food categories, and the HCC disease burden should remain a concern in future research efforts.

## 1. Introduction

Aflatoxins (AFs) are toxic compounds produced as secondary metabolites by the fungi species *Aspergillus flavus* and *Aspergillus parasiticus* [1]. Among all known categories of AF, the most commonly found in food and feed crops are aflatoxin B_1_ (AFB_1_), AFB_2_, AFG_1_, and AFG_2_ [2]. Additionally, AFM_1_, a hydroxylated derivative of AFB_1_, primarily occurs in milk and dairy products and is produced by the metabolism of lactating animals that consume AFB_1_-contaminated feed crops [3]. Chronic exposure to low doses of AF through diet can result in chronic toxicity, and AF can act as endogenous genotoxic agents, persistently stimulating carcinogenesis [4]. The International Agency for Research on Cancer (IARC) has classified AFB_1_, AFB_2_, AFG_1_, and AFG_2_ as Group 1 human carcinogens, indicating sufficient evidence of their carcinogenicity to humans [5]. AFB_1_ is the most potent carcinogenic aflatoxin and is a known cause of hepatocellular carcinoma (HCC). Its toxic mechanism involves the metabolite AFB_1_-8,9-epoxide, which binds to DNA in target cells, forming guanine adducts that lead to cell damage, gene mutations, and tumor formation [6]. Human exposure to AF combined with hepatitis B virus (HBV) infection greatly increases the risk of HCC, with an odds ratio (OR) of 54.1, compared to 5.91 for AF exposure alone and 11.2 for chronic HBV infection alone [7].

AF can contaminate various crops, including grains, beans, nuts, oilseeds, spices, and dairy products, with corn and peanuts being particularly susceptible. These food items are sources of human dietary exposure to AF [8]. While AF-producing fungi are widespread, contamination is more common in tropical and subtropical regions (16° to 35° latitude), where warm and humid climates promote fungal growth and AF production [9]. Moreover, in developing countries, AF contamination has become a severe social problem due to lower levels of economic development and the inadequate implementation of food safety standards. This is also a major global food safety issue [10]. The Joint FAO/WHO Expert Committee on Food Additives (JECFA) and the European Food Safety Authority (EFSA) have conducted a number of assessments on AF contamination in food and its impact on human health [11,12,13,14,15,16,17,18,19,20,21]. EFSA considers the liver carcinogenicity of AF as a pivotal consideration for assessing risks in both animals and humans. Many countries and regions have assessed the health risks of dietary AF exposure. Globally, AF exposure is responsible for 4.6–28.2% of liver cancer cases, with the highest rates in Sub-Saharan Africa, Southeast Asia, and China, ranging from 12.1% to 28.9% [22]. Additionally, research indicated that with an intake of 1 ng consumed per kilogram body weight per day, AFM_1_ could cause 13–32 cases of HCC per 100,000 people annually worldwide [23]. For AFB_2_, AFG_1_, and AFG_2_, the available in vivo data are not sufficient to derive carcinogenic potency factors, so EFSA applied equal carcinogenic potency factors for them as AFB_1_ [21]. Specially, under a dietary AFB_1_ exposure of 1 ng/kg bw/day, there would be 0.3 cases of HCC annually among 100,000 HBsAg-positive (HBsAg^+^) individuals and 0.01 cases for HBsAg-negatives (HBsAg^−^) individuals [13]. For AFM_1_, JECFA concluded, based on a study in Fischer rats, that AFM_1_ induces liver cancer with a potency one-tenth that of AFB_1_ [14].

Disease burden refers to the impact of diseases, injuries, and premature deaths on societal health and economies. The disability-adjusted life year (DALY) is a critical metric for assessing the loss of healthy life due to illness or injury [24,25]. The World Health Organization (WHO) has reported that global AF exposure leads to an estimated 21,757 cases of HCC, with 19,455 deaths and 636,869 DALY [26]. In China, however, there is limited knowledge about the burden of HCC from dietary AF exposure, as most researchers have focused on exposure risk without evaluating the related disease burden. For example, Li et al. assessed AF in cereals and oils in the Yangtze Delta, and Ding et al. examined AFB_1_ in peanuts after harvest in the Yangtze River’s ecological region, focusing only on exposure risk [27,28]. China spans over 20 degrees of latitude and has a diverse climate, encompassing five distinct climatic zones. A study has shown that regions in China with a high incidence of liver cancer generally have warm and humid climates [29]. Chen et al. estimated the DALY due to dietary AF intake in different regions of China (including Sichuan), but this study focused on only two food categories, peanuts and peanut oil, as well as corn and its products, which may lead to an underestimation of the overall foodborne aflatoxin-related disease burden for the target population [30]. In 2023, we estimated the disease burden of HCC due to ∑_4_AF (the sum of AFB_1_, AFB_2_, AFG_1_, and AFG_2_) exposure from three food categories among the population of Chongqing municipality, a city adjacent to Sichuan Province in southwest China with a subtropical monsoon climate.

Various studies have highlighted the health risks posed by AF in grains, nuts, oilseeds, and spices [31,32]. These foods are commonly consumed in Sichuan, a major grains-producing region located between 26° N and 34° N with a subtropical monsoon climate that promotes fungal growth and toxin production [33,34]. According to the latest GLOBOCAN estimates produced by the IARC, the age-standardized incidence rate by world standard population of liver cancer in the Chinese population is significantly higher than the global level (15.0 cases per 100,000 persons in China, compared to 8.6 cases per 100,000 persons globally) [35,36]. According to Chinese cancer registry data, the age-standardized incidence rate by the Chinese standard population of liver cancer in the Sichuan population is higher than the national level in China (17.75 cases per 100,000 persons in Sichuan, compared to 9.71 cases per 100,000 persons in China) [37,38]. Despite this, no studies have yet assessed the health risks and corresponding HCC disease burden from exposure to ∑_5_AF (the sum of AFB_1_, AFB_2_, AFG_1_, AFG_2_, and AFM_1_) among the population of Sichuan. Therefore, estimating the HCC disease burden from dietary exposure to ∑_5_AF in Sichuan is crucial to provide more evidence for the risk management of AF in this area.

## 2. Materials and Methods

### 2.1. Sample Collection

Based on the sampling guidelines of the China National Food Safety Risk Monitoring Program and excluding food categories with less than 10 samples, a total of 4359 samples were collected from 2012 to 2023, covering all cities and prefectures in Sichuan Province. The collected food types mainly included those commonly consumed by the population of Sichuan and potentially susceptible to AF contamination. Food categories included grains and their products (rice and its products, corn and its products, wheat and its products, and coix seed), condiments (spices, hot-pot seasoning, vinegar, sauce and its products, and soy sauce), nuts and seeds (peanut and its products, other nuts, and seeds), milk and dairy products, beans and their products, foods for special dietary use (infant formula and complementary cereals for infants), plant-based protein drinks, vegetable oil (peanut oil and corn oil as well as other vegetable oil), puffed food (based on corn, such as popcorn, puffed corn chips, corn crisps, and puffed corn balls), beer, and tea leaves.

### 2.2. Sample Analysis

According to the China National Food Safety Standards (GB/T 18979-2003 [39], GB/T 5009.23-2006 [40], GB 5413.37-2010 [41], and GB 5009.22-2016 [42]), the samples were analyzed using high-performance liquid chromatography (HPLC) or HPLC coupled with tandem mass spectrometry. AFB_1_, AFB_2_, AFG_1_, and AFG_2_ were analyzed in food categories excluding infant formula, milk, and dairy products, which only analyzed AFM_1_. AF standard substances and isotope-labeled internal standard substances were used as certified reference materials. All chemicals and solvents used were of analytical grade or higher, and participating laboratories were provincially accredited, ensuring that they met the quality control requirements for testing. The aflatoxin contamination data used in this study were obtained from the testing laboratories of all municipal-level Centers for Disease Control and Prevention in Sichuan Province. Due to advances in detection methods over the past decade (from chromatography to mass spectrometry) and differences in instrument sensitivity across laboratories, the limits of detection (LOD) varied. The LOD for AFB_1_ was 0.001–10 μg/kg, and for AFB_2_, AFG_1_, and AFG_2_, it was all 0.001–5 μg/kg, and for AFM_1_, it was 0.0002–0.1 μg/kg. All LODs met the required standards of the methods used.

### 2.3. Data Processing

In adherence to the Global Environment Monitoring System/Food Contamination Monitoring and Assessment Program’s principles for handling undetected values, these values were assigned either 0 for lower bound (LB) estimates or LOD for upper bound (UB) estimates [43]. ∑_4_AF (the sum of AFB_1_, AFB_2_, AFG_1_, and AFG_2_) was calculated based on the principles of the EFSA report in 2020 [15]. JECFA pointed out that the carcinogenic potency of AFM_1_ is one-tenth that of AFB_1_. Therefore, the exposure level of ∑_5_AF for the target population was calculated using the ∑_4_AF contamination level and one-tenth of the AFM_1_ contamination level [14,19,20,21].

### 2.4. Consumption Data

The consumption data in Sichuan Province were mainly obtained from the 2011 China National Nutrition and Health Survey [44,45]. A multi-stage stratified and population-proportional cluster random sampling method was used in this survey to form a nationally representative dietary nutrition and health database. Dietary consumption data for respondents aged 2 and above were collected via 3 consecutive 24 h dietary recalls through in-person interviews. After data cleaning and sorting, the consumption data of 2836 respondents aged 2 years and above were obtained from Sichuan Province. Since the consumption data of respondents under 2 years old were excluded in the above survey, our study estimated the consumption of formula and complementary cereals for infants under 2 years old by referring to the data from the 2015 China National Food Consumption Survey reported in the literature [46,47]. For each individual surveyed, the total food consumption in each category was aggregated and aligned with the contamination data for the corresponding food categories.

### 2.5. Estimation Method

**Figure 1:**
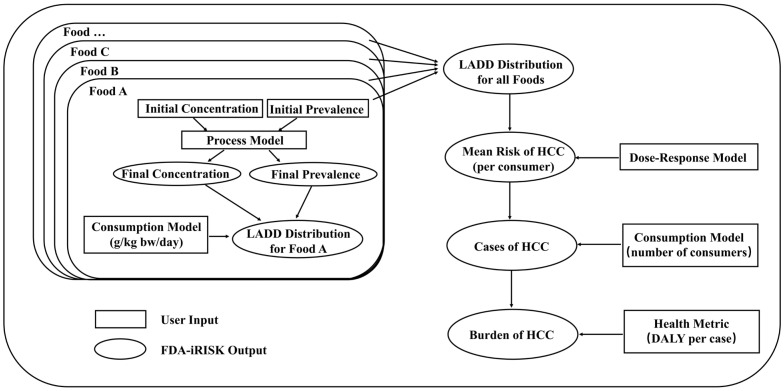
Technical route for assessing chronic exposure to AF in multiple food used in iRISK.

FDA-iRISK 4.2 (iRISK), comprising four pivotal components: hazard, food, process, and risk scenario modules, was utilized to estimate lifetime chronic dietary exposure to ∑_5_AF and corresponding HCC burden [48]. Figure 1 shows the technical route.

#### 2.5.1. Lifetime Exposure Assessment

##### Consumption Model

In iRISK, food consumption patterns were defined using a linear empirical distribution to account for varying consumption rates across life stages, including a portion of the population with zero consumption [49]. For infant formula and complementary cereals, a single average consumption value was assumed with a 100% probability for infants under 2 years old. Population groups were categorized by life stages, based on average life expectancy, as shown in Supplementary Table S1. Individuals exceeding this age were included in the “66-average life expectancy” group.

##### Process Model

The process model assessed how different interventions throughout the “farm-to-table” process influenced AF contamination level in food and then calculated the ∑_5_AF exposure for the target population by integrating the hazard and food modules. Since food samples were homogenized and not cooked prior to analysis, AF concentrations can be considered uniformly distributed within the food and assumed to remain constant throughout the process. This means that the proportion of AF contamination per unit mass of food was 100% before and after conducting the process model (initial and final prevalence were both 100%) [50]. This study found that varying the set value of unit mass, defined as 1.0 kg, did not impact the contamination levels of AF in food. Supplementary Table S1 shows these input parameters. Each subject was assumed to maintain the same type of diet throughout their lives as declared during the three days considered in the interview. Any variations in their diet predicted by iRISK were only related to age group, which were associated with variations in body weight and the quantity of food consumed. Additionally, the AF contamination levels of foods consumed by the subjects throughout their lives were assumed to be same as that measured during this study.

The lifetime average daily dose (*LADD*) to the ∑_5_AF intake across all studied food categories was calculated according to the following formulas:

(1) $$LADD=\sum_{k=1}^{m}{LADC}_{k}\times C_{k}\times 100\%=\sum_{k=1}^{m}{LADD}_{k}$$

(2) $${LADC}_{k}=\sum_{i=1}^{n}A_{i}\times Y_{i}/L$$

where *LADC_k_* is the lifetime average daily consumption of food category *k* (g/kg bw/day). Food categories include rice and its products, corn and its products, wheat and its products, coix seed, peanut and its products, other nuts and seeds, spices, vinegar, sauce and its products, soy sauce, peanut oil and corn oil, other vegetable oil, beans and their products, milk and dairy products, plant-based protein drinks, beer, puffed food, tea leaves, infant formula, and complementary cereals for infants. *LADD_k_* is the LADD of ∑_5_AF from food category *k* (ng/kg bw/day); *C_k_* is the final AF content in food category *k* (μg/kg); 100% is the final AF prevalence; *m* is the number of food categories; *A_i_* is the daily consumption per kg bw for life stage *i* (g/kg bw/day); *Y_i_* is the duration of life stage *i* (years); *n* is the number of life stages; and *L* denotes the average life expectancy. According to the Sichuan Population Health and Key Diseases Report of 2022, the average life expectancy is 77.91 years (75.26 years for males and 80.99 years for females) [51]. The calculation methodology and illustrative examples of the LADD are detailed in Supplementary Tables S2 and S3.

#### 2.5.2. HCC Disease Burden Estimation

##### Dose–Response Model

To quantify the relationship between AF exposure and the incidence of HCC, the cancer slope factor (SF), derived from JECFA cancer potency factor (PF), was employed as a metric. JECFA estimated that for a dietary AFB_1_ exposure of 1 ng/kg bw/day, there would be 0.3 cases of HCC annually among 100,000 HBsAg^+^ individuals and 0.01 cases for HBsAg^−^ individuals [13]. According to EFSA and JECFA, the carcinogenic potency of AFB_2_, AFG_1_, and AFG_2_ was assumed to be the same as that of AFB_1_, and the carcinogenic potency of AFM_1_ was one-tenth that of AFB_1_ [14,19,20,21]. Therefore, the carcinogenic potency of ∑_5_AF (the sum of AFB_1_, AFB_2_, AFG_1_, AFG_2_, and one-tenth of AFM_1_) was conservatively assumed to be the same as that of AFB_1_ in our study.

The *SF* [50] and *PF* were calculated according to the following formulas:

(3) SF=1÷(100,000 persons×10%/PF/L)

(4) $$PF=P_{HBsAg+}\times 0.3+\left(1-P_{HBsAg+}\right)\times 0.01$$

where 10% is a 10-percentage-point elevation in the lifetime hepatocellular carcinoma risk [50]. The calculated SF is shown in Supplementary Table S1. *P_HBsAg+_* represents the HBsAg^+^ rate of 1.29% for the population of Sichuan, as reported in the China National Seroepidemiological Survey on Hepatitis B in 2014 [52].

##### DALY Estimation

Due to the inclusion of subjects with zero food consumption in the consumption distribution, it is possible that not all 20 food types were consumed at each life stage in the exposure pattern simulation. Therefore, the simulation of the lifetime chronic exposure patterns considered both qualitative and quantitative patterns. iRISK estimated a large number of LADD values by simulating various individual lifetime chronic exposure patterns within the consumers. The distinct variants of LADD were consolidated into a representative value (LADD_R_). The dose–response model was then used to calculate the average HCC risk for each consumer.

This study utilized DALY as a metric to estimate the HCC disease burden attributable to foodborne AF. According to the 2019 China Cancer Registry Annual Report, the DALY per HCC case in China was calculated to be 12.37 [30,53]. To calculate the total DALYs caused by foodborne ∑_5_AF, the number of HCC cases related to foodborne ∑_5_AF was multiplied by a factor of 12.37.

The lifetime DALY of the target population (*DALYs*) was calculated according to the following formulas:

(5) $${DALY}_{s}=12.37\times N$$

(6) N=P×M

(7) $$P={LADD}_{R}\times SF\times 100\%$$

where *N* is the number of HCC cases; *P* is the average probability of developing HCC over a lifetime for an individual consumer (%); *M* is the number of target populations (consumers). According to the major figures in the 2020 Population Census of China, the population of Sichuan Province consists of 8,367,000 individuals, with males at 50.54% and females at 49.46% [54].

##### DALY Rate Calculation

DALY rate was employed to quantify the annual burden of HCC attributable to dietary ∑_5_AF intake among 100,000 persons. Taking into account the number of individuals in the target population and the average life expectancy, the *DALY rate* (DALY/100,000 persons/year) was calculated as follows:

(8) $$DALY\;rate={DALY}_{s}\times 100,000/M/L$$

where *L* is the average life expectancy.

##### Population Attributable Fraction Calculation

Population attributable fraction (PAF) was employed to estimate the proportion of HCC cases in Sichuan that is attributable to ∑_5_AF intake. The PAF was calculated as follows:

(9) PAF=N×100,000×100%/M/L/R

where *L* is the average life expectancy; *R* is the annual all-cause liver cancer incidence (cases/100,000 persons/year). According to the latest 2019 cancer registry data of Sichuan Province, the incidence of all-cause liver cancer in the whole population in Sichuan Province was 31.21 cases/100,000 persons/year, with 46.63 cases/100,000 persons/year in males and 15.04 cases/100,000 persons/year in females [37].

### 2.6. Statistical Analysis

Statistical analysis was performed using SPSS 25.0 [55]. Since the AF contamination data did not follow a normal distribution, the values were represented by the mean and the percentiles. The lifetime chronic dietary exposure levels of ∑_5_AF and the corresponding disease burden of HCC were assessed using FDA-iRISK 4.2 [49].

## 3. Results

### 3.1. AF Contamination Levels

As listed in Table 1, the rate of ∑_4_AF detection in food from Sichuan was 10.4%, with high detection rates primarily found in grains and their products, condiments, and plant-based protein drinks. Hot-pot seasoning had the highest detection rate at 42.9%, followed by plant-based protein drinks at 32.3%. In terms of the mean concentration of ∑_4_AF in various food categories, corn and its products had the highest levels (5.25–6.00 μg/kg, LB-UB), approximately 2–3 times that of the total samples. The max concentration level of ∑_4_AF in corn and its products was up to 865.0–865.2 μg/kg, followed by peanut and its products (318.0 μg/kg). Although hot-pot seasoning and plant-based protein drinks had higher ∑_4_AF detection rates than all other food categories, their ∑_4_AF concentrations were relatively low. Regarding AFM_1_ detection, none of the 143 infant formula samples were detected positive, and only 2 out of 525 milk and dairy product samples were detected, with not significantly contaminated.

### 3.2. Food Consumption Data

As shown in Table 2 and Table 3, grains and their products were the main food category in the diet of the respondents aged 2 years and above in Sichuan. Among these, rice and its products had the lowest proportion of non-consumers (1.9%) and the highest average consumption (2.26 g/kg bw/day), followed by wheat and its products (9.3%, 1.55 g/kg bw/day). However, more than 99% of the respondents aged 2 years and above in Sichuan did not consume coix seed, sauce and its products, plant-based protein drinks, puffed food, and tea leaves, with the average consumption of these foods being close to 0 g/kg bw/day. In Table 4, the consumption of infant formula and complementary cereals for infants under 2 years old was based on the reported data from the 2015 China National Food Consumption Survey. This study assumed that the proportion of non-consumers of the above baby foods in this age group was 0%.

### 3.3. Lifetime Dietary Exposure to ∑_5_AF

**Figure 2:**
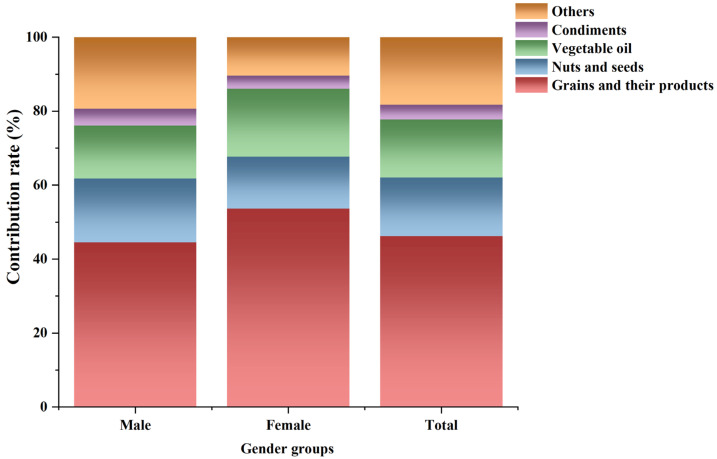
Contribution rate of various food categories to lifetime dietary exposure to ∑_5_AF in Sichuan Province, China.

As listed in Table 5, at both the mean and high (P_95_) AF contamination levels in food, the LADD_R_ for total population in Sichuan ranged from 9.77 (LB) to 26.0 (UB) ng/kg bw/day and from 22.1 to 85.5 ng/kg bw/day, respectively. Regardless of whether AF contamination levels in food were at the mean or high, the LADD_R_ for males (9.44–25.0 ng/kg bw/day and 20.6–83.4 ng/kg bw/day) in Sichuan was higher than that of females (8.74–21.2 ng/kg bw/day and 18.6–61.3 ng/kg bw/day). For the percentile values of LADD, except for the P_95_ and P_99_ values of LADD under the LB estimate of the average AF contamination level and the P_95_ value of LADD under the LB estimate of the high AF contamination level, the LADD values for males were higher than those for females in all other cases. Except for the median LADD of the total population under the LB estimate of the average AF contamination level, which lies between the median LADD values of females and males, in all other cases, the total population LADD is higher than the LADD values for both males and females. As shown in Figure 2, among all food categories, grains and their products contributed the most to dietary exposure to ∑_5_AF for the whole population of Sichuan Province, accounting for 46.2%. Specifically, the contribution rate was 44.5% for males but exceeded 50% for females.

### 3.4. HCC Disease Burden of Lifetime ∑_5_AF Exposure

As shown in Table 6, at the mean level of AF contamination, the LADD_R_ led to an estimated 89,100–237,000 HCC cases in Sichuan, causing 41,900–111,000 HCC cases in males and 40,900–99,200 HCC cases in females. In addition, the lifetime risk of HCC per person ranged from 0.106% to 0.283%, with the total number of corresponding DALYs, DALY rate, and PAF estimated to be 1,100,000–2,930,000, 16.87–44.95/100,000 persons/year, and 4.4–11.6% for the entire population, respectively. In the same scenario, the lifetime risk of both HCC and DALYs for males in Sichuan was higher than those for females. However, PAF was higher in females (8.1–19.7%) than in males (2.8–7.5%) because the incidence of all-cause liver cancer was significantly lower in females (15.04 cases/100,000 persons/year) than in males (46.43 cases/100,000 persons/year). Notably, compared to the HCC disease burden associated with mean contamination levels, lifetime dietary exposure to extreme cases (where ∑_5_AF contamination levels reach P_95_ in all food categories studied) resulted in a 2–3 times higher HCC disease burden.

## 4. Discussion

This study is the first to simulate lifetime dietary chronic exposure patterns to ∑_5_AF across 20 food categories in Sichuan Province, China, and estimate corresponding HCC disease burden. Under the mean AF contamination level, the LADD_R_ of dietary exposure to ∑_5_AF in Sichuan was similar to the results of 24.9 ng/kg bw/day for adults from a study assessing dietary AF exposure in the Yangtze River Delta region of China. However, the high (P_95_) exposure levels (49.8 ng/kg bw/day) were higher than our estimates, with the AF exposure in that study coming solely from grains and their products [28]. The discrepancy may be attributed to the higher mean and P_95_ daily grain consumption figures used in our calculations, which were derived from per capita daily intake values of 402 g and 804 g for adults and children. In 2023, we estimated the ∑_5_AF exposure from three food categories in Chongqing, China, to be 2.4–8.25 ng/kg bw/day [56], lower than the ∑_5_AF exposure in Sichuan Province due to fewer food categories (only three) and AF types (AFB_1_, AFB_2_, AFG_1_, and AFG_2_) included. Asian comparisons revealed significantly lower AFB_1_ exposure in Korea and Japan compared to Sichuan. Korean exposure ranged from 0.0640 to 0.3612 ng/kg bw/day, while Japan’s heavy consumers’ exposure was merely 0.003–0.004 ng/kg bw/day [57,58]. However, in India, the average AF intake solely through rice consumption for children, adolescents, and adults reached 18.55, 13.09, and 12.32 ng/kg bw/day, respectively. These figures are close to the estimates of lifetime dietary exposure to ∑_5_AF from 20 food categories in the population of Sichuan. The reason may be due to the higher mean AF contamination in rice (1.92 μg/kg) and the higher rice consumption in India, with adults consuming 386 g/person/day, adolescents 328 g/person/day, and children 184 g/person/day [59].

In the EFSA’s 2020 report, the estimated ranges of average and P_95_ exposure to ∑_5_AF from multiple foods for different age groups in Europe were 0.42–9.14 ng/kg bw/day and 0.95–16.08 ng/kg bw/day, respectively, both lower than in Sichuan [15]. This discrepancy may be related to the fact that AF contamination received more attention, as most regions in Europe have a temperate climate, whereas most regions in Sichuan have a subtropical climate. Multiple studies have indicated that AF exposure levels are higher in Sub-Saharan Africa, Southeast Asia, and China, as these regions are in tropical or subtropical zones where climatic conditions favor AF contamination in foods [60,61]. For instance, Sub-Saharan Africa experiences significant exposure, with AFB_1_ estimates spanning from 4 to 526 ng/kg bw/day [62]. A recent Accra study highlights even higher aflatoxins intakes, estimating 436 ng/kg bw/day from maize consumption and 63.2 ng/kg bw/day from peanut consumption [63].

The iRISK calculates the HCC disease burden due to lifetime chronic dietary exposure to ∑_5_AF from multiple foods for population in Sichuan. This result contrasts significantly with a 2022 study in China, which reported a national average DALY rate of 1.53 DALY/100,000 persons/year caused by dietary exposure to ∑_5_AF and a much lower DALY rate of only 0.07 DALY/100,000 persons/year in Sichuan. This significant difference may stem from the previous study’s focus solely on AF in peanuts within Sichuan, estimating an exposure level of just 0.138 ng/kg bw/day. However, our results indicated that the AF contamination levels in grains and their products, as well as the consumption of grains and their products in the population, were both the highest among 20 food categories in Sichuan. Hence, grains and their products are the primary dietary sources of AF exposure for the population in Sichuan [30]. Taiwan of China found that AF contamination resulted in 4110 DALY caused by HCC annually for the Taiwanese population, translating to 24.63 DALY/100,000 persons/year. Although this result was lower than the corresponding DALY rate for Sichuan, the Taiwanese study only assessed the AF exposure risk from peanuts, and the HBsAg^+^ rate in Taiwan (17.3%) was much higher than in Sichuan (1.29%) [50].

Globally, this study found Sichuan’s DALY rate related to AF intake to be higher than WHO’s 2015 median estimates (9 DALY/100,000 persons/year). Among the 14 sub-regions, the highest median DALY rate was in the Africa D region at 28 DALY/100,000 persons/year, while the Western Pacific B region (which includes China) had a median of 17 DALY/100,000 persons/year. Both of these DALY rates are lower than the estimates from this study. The regions with the lowest DALY rates were the Americas A region and the Europe A region, with medians of 0.04 DALY/100,000 persons/year and 0.3 DALY/100,000 persons/year, respectively [26]. The WHO’s estimate of the foodborne AF burden was based on counterfactual analysis, which might lead to differences from the dose–response method used in our study. The global number of HCC cases attributable to AF was estimated to be 22,000 (9000–57,000) annually using counterfactual analysis, whereas using a dose–response approach, it was estimated to be 25,200–15,500. Although there was overlap between the two ranges, the differences were still significant. The WHO suggested that the dose–response method might overestimate the disease burden, while the global HCC disease burden might be underestimated, particularly in Africa [64]. At the national level, the HCC disease burden due to dietary AF exposure in the Portuguese population was only 0.08–0.30 DALY/100,000 persons /year, and that from maize alone in the Nigerian population (126.85–38,682.29 DALY/100,000 persons /year) was far higher than in Sichuan [65,66]. Based on the demographic data from the UN’s World Population Prospects 2022, the HCC disease burden from AF exposure in Tanzanian maize and peanuts (5.61–280.5 DALY/100,000 people/year) was much higher than in Sichuan, as maize and peanuts were the most important crops in Tanzania, with maize accounting for 40% of the total caloric intake of Tanzanian households [67]. The above findings were consistent with the WHO’s conclusion that the foodborne AF disease burden was highest in the African region.

Based on the AF contamination in Sichuan from 2012 to 2023, hot-pot seasoning exhibited the highest detection rate (42.9%). This could be attributed to the fact that the raw materials for hot-pot seasoning encompass a diverse array of spices such as chili peppers, Sichuan peppers, star anises, cinnamon, bay leaves, and cumin seeds, among others [68]. These spices are prone to mold growth and toxin production during the food production process, ultimately leading to AF contamination in the final products. Notably, the detection rate of AFB_1_ in commercially available hot-pot seasoning in Guizhou Province, China, also reached a substantial level of 40% [69]. The AF detection rate in plant-based protein drinks from Sichuan (32.3%) ranked second, likely due to their raw materials. These drinks, made primarily from nuts like peanuts, walnuts, and almonds, are highly susceptible to AF contamination [70]. Apart from nuts, grains, particularly corn and its products, also constituted a significant source of AF contamination in Sichuan. Among nuts, peanut and its products exhibit the highest levels of AF contamination. As both of these crops are grown in tropical climates with temperatures ranging from 25 to 35 °C, with high water content and rich nutrition, they are particularly vulnerable to fungus development and AF contamination [71]. According to the data of AF contamination in food from 2012 to 2023 in this study, the AF contamination level in food in Sichuan Province had no decreasing trend, especially in corn and spices, but there was an obvious rising trend. As future climate change may increase AF contamination in food, effective measures are needed to reduce dietary AF exposure and lower the HCC disease burden. Vigilant monitoring and control throughout the food production chain are essential to mitigate AF contamination in high-risk food categories.

Although this study was the first to use the iRISK to simulate the lifetime chronic dietary exposure to ∑_5_AF in Sichuan and quantify the HCC disease burden, several uncertainties remain. First, due to a lack of research data on cancer potency factors for all AF types, this study applied the carcinogenic of AFB_1_ to ∑_5_AF. While this conservative assumption introduced some uncertainty, EFSA has suggested it minimally impacts health risk conclusions for dietary AF exposure [12]. Secondly, the consumption data for the population aged 2 years and above were obtained from the 2011 China National Nutrition and Health Survey, while the estimated consumption data for those under 2 years old were obtained from the data of the 2015 China National Food Consumption Survey reported in the literature. Dietary patterns may have shifted over the past decade due to socio-economic changes. Furthermore, the AF contamination data used in this study spanned more than ten years (2012–2023). This dual temporal misalignment and the difference in recruitment between the two sets of consumption data may have introduced certain uncertainties in the results. The 3-day, 24 h recall method used in the China National Nutrition and Health Survey relies on respondents’ accurate recall of their dietary intake. The Chinese Center for Disease Control and Prevention developed a unified quality control plan for the survey, which includes standardized questionnaires and interviewer training to ensure data accuracy. Furthermore, the survey design includes multiple days of data collection (3 consecutive days) to account for variations in individual eating patterns, thus reducing bias from a single day’s recall. In addition to the 3-day recall, the survey also includes food frequency questionnaires, which help mitigate potential bias from relying on a single method. Additional uncertainties arise from the iRISK simulation itself. In the consumption model, since the food consumption data for infants under 2 years old were estimated as a single value, it was impossible to construct a complete food consumption distribution for this group, introducing uncertainty into the overall food consumption distribution, model iterations, and final exposure estimates across all life stages. Each subject was assumed to follow the same diet throughout their life as declared during the interview. It was also assumed that the AF contamination levels in their diet throughout their life would remain constant as measured in this study. The samples in this study were primarily collected from markets. The iRISK process module assumed that AF content in the food remained unchanged throughout processing. However, different food preparation (washing, peeling, etc.) and cooking methods (steaming, frying, baking, etc.) can reduce AF levels in foods to varying degrees [72,73,74], and improper storage of foods after purchase could potentially increase AF levels. Due to the lack of data in this study to assess the extent of change in AF levels during food preparation and cooking after food procurement, this study follows risk assessment principles and assumes transparency, considering such factors only as uncertainties acknowledged as a limitation in our study and discussed as such. Therefore, the above assumptions introduced uncertainties that could not be quantified into this study. Future studies should comprehensively assess the AF contamination process to minimize uncertainties and gain a more accurate understanding of the HCC disease burden from lifetime chronic dietary exposure to total AF.

## 5. Conclusions

Based on Sichuan Province’s demographics, dietary patterns, and AF contamination levels in vulnerable foods, the population’s lifetime exposure to ∑_5_AF results in an HCC burden higher than the global level. Additionally, while males had a higher risk of developing HCC, the PAF was higher in females. Among the studied food categories, grains and their products contributed the most to AF exposure in Sichuan population. Given the potential for future climate change to increase AF contamination in food, it is recommended to continuously monitor and control AF contamination, particularly in highly vulnerable food categories. Additionally, the disease burden of HCC attributed to lifetime chronic exposure to AF should also be a focus of further research. This will help reduce dietary exposure to total AF among the population, thereby mitigating the HCC disease burden at its source.

## Figures and Tables

**Table 1:** ∑_4_AF contamination levels (μg/kg) across various food categories in Sichuan Province, China.

Food Category	N	Positive Samples (%)	Mean		Median		P95		Max	
			LB ^1^	UB ^2^	LB	UB	LB	UB	LB	UB
Grains and their products										
Rice and its products	324	23 (7.1)	0.35	0.57	0.00	0.12	0.36	1.60	70.80	70.92
Corn and its products	965	132 (13.7)	5.25	6.00	0.00	0.40	8.48	10.00	865.0	865.2
Wheat and its products	263	0 (0.0)	0.00	0.48	0.00	0.60	0.00	1.00	0.00	1.00
Coix seed	79	17 (21.5)	0.71	1.05	0.00	0.40	5.13	5.33	19.13	19.53
Nuts and seeds										
Peanut and its products	328	60 (18.3)	2.75	3.47	0.00	0.20	5.53	10.00	318.00	318.00
Others	425	17 (4.0)	0.10	0.87	0.00	1.00	0.00	2.00	11.80	11.86
Condiments										
Spices	340	62 (18.2)	0.35	0.73	0.00	0.20	0.78	3.00	28.15	28.35
Hot-pot seasoning	21	9 (42.9)	0.05	0.18	0.00	0.20	0.26	0.46	0.27	0.47
Vinegar	16	0 (0.0)	0.00	0.25	0.00	0.25	0.00	N/A ^3^	0.00	0.30
Sauce and its products	72	13 (18.1)	0.22	0.37	0.00	0.16	1.58	1.64	5.11	5.17
Soy sauce	28	2 (7.1)	0.04	0.15	0.00	0.10	0.51	0.61	0.54	0.64
Vegetable oil										
Peanut oil and corn oil	68	5 (7.4)	0.02	1.96	0.00	1.28	0.20	6.00	0.40	6.00
Others	154	0 (0.0)	0.00	1.67	0.00	1.28	0.00	4.00	0.00	4.00
Beans and their products	55	2 (3.6)	0.03	0.67	0.00	0.10	0.16	3.00	0.82	3.00
Complementary cereals for infants	60	2 (3.3)	0.01	0.28	0.00	0.20	0.00	0.48	0.24	0.64
Plant-based protein drinks	96	31 (32.3)	0.06	0.07	0.00	0.02	0.28	0.30	1.34	1.36
Beer	20	1 (5.0)	0.04	1.29	0.00	0.06	0.70	8.00	0.74	8.00
Puffed food	48	3 (6.3)	0.03	0.03	0.00	0.20	0.26	0.32	0.90	1.10
Tea leaves	329	4 (1.2)	0.03	0.69	0.00	0.50	0.00	2.00	2.39	2.39
Total	3691	383 (10.4)	1.92	2.56	0.00	0.20	0.95	3.00	865.00	865.20

^1^ Lower bound estimate: the non-detected results were assigned to 0. ^2^ Upper bound estimate: the non-detected results were assigned to LOD. ^3^ Not available.

**Table 2:** Percentage (%) of non-consumers for various food categories in Sichuan Province, China.

Food Category ^1^	2–6 Years Old		7–17 Years Old		18–65 Years Old		>65 Years Old		Total
	Male	Female	Male	Female	Male	Female	Male	Female	
Grains and their products									
Rice and its products	8.3	3.1	0.0	2.2	1.1	1.9	3.1	2.4	1.9
Corn and its products	97.2	100.0	93.3	94.6	95.0	93.6	92.3	93.3	94.1
Wheat and its products	8.3	9.4	10.5	14.1	8.4	8.9	11.2	9.9	9.3
Coix seed	100.0	100.0	100.0	100.0	99.4	99.7	97.7	99.2	99.4
Nuts and seeds									
Peanut and its products	97.2	96.9	98.1	97.8	93.4	93.5	93.4	95.6	94.1
Others	94.4	100.0	98.1	96.7	97.6	94.5	95.8	97.6	96.2
Condiments									
Spices	47.2	46.9	50.5	50.0	62.7	62.9	68.3	68.7	62.6
Vinegar	55.6	78.1	59.0	64.1	47.0	47.0	45.6	43.3	48.0
Sauce and its products	100.0	100.0	99.0	100.0	99.4	99.6	98.8	99.2	99.4
Soy sauce	16.7	21.9	20.0	23.9	14.8	15.5	21.2	16.7	16.4
Vegetable oil									
Peanut oil and corn oil	97.2	93.8	93.3	97.8	97.3	97.0	96.1	97.6	96.9
Others	13.9	18.8	14.3	14.1	10.3	11.1	13.5	12.3	11.5
Beans and their products	61.1	62.5	52.4	53.3	58.5	60.2	54.4	52.8	58.0
Milk and dairy products	58.3	59.4	61.9	52.2	90.5	88.6	83.8	87.7	85.9
Plant-based protein drinks	97.2	87.5	100.0	100.0	99.4	99.7	98.8	99.6	99.4
Beer	100.0	100.0	99.0	100.0	97.8	99.5	99.2	100.0	98.9
Puffed food	100.0	100.0	98.1	98.9	99.6	99.5	99.6	99.2	99.5
Tea leaves	100.0	100.0	99.0	98.9	99.2	99.5	97.3	99.2	99.1

^1^ Zero consumption of these food categories was assumed to be 100% for infants under 2 years old in Sichuan Province, China.

**Table 3:** Average daily consumption levels (g/kg bw/day) for various food categories among the consumers of Sichuan Province, China.

Food Category ^1^	2–6 Years Old		7–17 Years Old		18–65 Years Old		>65 Years Old		Total
	Male	Female	Male	Female	Male	Female	Male	Female	
Grains and their products									
Rice and its products	2.67	2.97	3.16	2.65	2.32	2.10	2.24	2.04	2.26
Corn and its products	0.01	0.00	0.05	0.03	0.03	0.04	0.03	0.04	0.04
Wheat and its products	1.93	2.05	1.56	1.38	1.64	1.51	1.47	1.43	1.55
Coix seed	0.00	0.00	0.00	0.00	0.00	0.00	0.01	0.00	0.00
Nuts and seeds									
Peanut and its products	0.02	0.01	0.01	0.02	0.04	0.02	0.03	0.01	0.03
Others	0.03	0.00	0.01	0.00	0.01	0.02	0.01	0.01	0.01
Condiments									
Spices	0.40	0.20	0.14	0.19	0.11	0.09	0.08	0.06	0.10
Vinegar	0.12	0.05	0.05	0.09	0.07	0.07	0.09	0.06	0.07
Sauce and its products	0.00	0.00	0.00	0.00	0.00	0.00	0.00	0.00	0.00
Soy sauce	0.32	0.29	0.16	0.19	0.15	0.15	0.12	0.13	0.15
Vegetable oil									
Peanut oil and corn oil	0.05	0.03	0.05	0.01	0.01	0.02	0.01	0.01	0.02
Others	0.84	0.95	0.91	0.68	0.77	0.71	0.63	0.62	0.73
Beans and their products	0.19	0.11	0.16	0.22	0.14	0.13	0.21	0.17	0.15
Milk and dairy products	3.62	2.83	1.64	2.57	0.24	0.27	0.41	0.41	0.48
Plant-based protein drinks	0.06	0.49	0.00	0.00	0.01	0.00	0.01	0.00	0.01
Beer	0.00	0.00	0.00	0.00	0.10	0.01	0.03	0.00	0.04
Puffed food	0.00	0.00	0.01	0.00	0.00	0.00	0.00	0.01	0.00
Tea leaves	0.00	0.00	0.00	0.00	0.00	0.00	0.00	0.00	0.00

^1^ Consumption of these food categories was assumed to be 0 g/kg bw/day for infants under 2 years old in Sichuan Province, China.

**Table 4:** Estimated consumption levels of two types of baby food in China (g/kg bw/day).

Age Group (Months) ^1^	Infant Formula	Complementary Cereals for Infants
0–6	5.19	0.27
7–12	5.01	0.74
13–24	4.73	0.26

^1^ Consumption of these two types of baby food was estimated using data from the 2015 China Food Consumption Survey, as reported in the literature by Wang et al. in 2019 and Li et al. in 2021 [46,47].

**Table 5:** Lifetime dietary exposure levels to ∑_5_AF for different groups in Sichuan Province, China (ng/kg bw/day).

Aflatoxin Contamination Level ^1^	Group	LADD_R_		Median		P_95_		P_99_	
		LB ^2^	UB ^3^	LB	UB	LB	UB	LB	UB
	Male	9.44	25.0	9.31	24.5	13.8	34.3	16.0	40.1
Mean	Female	8.74	21.2	7.88	20.3	15.2	30.0	16.8	34.9
	Total	9.77	26.0	9.14	25.3	15.8	36.1	18.2	41.9
	Male	20.6	83.4	20.3	81.3	29.1	118	33.2	132
P_95_ ^4^	Female	18.6	61.3	17.5	59.9	29.2	81.7	32.6	92.7
	Total	22.1	85.5	21.3	83.0	32.7	121	37.2	136

^1^ Due to the lack of consumption data of hot-pot seasoning and the fact that the main ingredients of hot-pot seasoning are spices such as chill pepper, Sichuan pepper, star anise, cinnamon, and so on, hot-pot seasoning was included in the “Spices” category for exposure calculation. ^2^ Lower bound estimate: the non-detected values in the contamination data were assigned 0 and then used to calculate the exposure. ^3^ Upper bound estimate: the non-detected values in the contamination data were assigned LOD and then used to calculate the exposure. ^4^ The UB of max value of ∑_4_AF contamination in vinegar which was not available was substituted with the corresponding P_95_ value.

**Table 6:** HCC disease burden attributable to the LADD_R_ of dietary exposure to ∑_5_AF in Sichuan Province, China.

Aflatoxin Contamination Level	Group	HCC Case (×10,000)		HCC Risk (%) ^1^		DALYs (×10,000)		DALY Rate (DALY/100,000 Persons/Year)		PAF (%)	
		LB ^2^	UB ^3^	LB	UB	LB	UB	LB	UB	LB	UB
	Male	4.19	11.10	0.0991	0.262	51.8	137	16.28	43.05	2.8%	7.5%
Mean	Female	4.09	9.92	0.0987	0.240	50.5	123	15.07	36.70	8.1%	19.7%
	Total	8.91	23.70	0.106	0.283	110	293	16.87	44.95	4.4%	11.6%
	Male	9.16	37.0	0.217	0.875	113	458	35.61	143.5	6.2%	24.9%
P_95_	Female	8.69	28.6	0.210	0.692	107	354	32.10	105.7	17.3%	56.8%
	Total	20.2	78.0	0.241	0.932	249	965	38.25	147.6	9.9%	38.2%

^1^ Lifetime risk of HCC per person. ^2^ Lower bound estimate: the non-detected values in the contamination data were assigned 0 and then used to calculate the disease burden. ^3^ Upper bound estimate: the non-detected values in the contamination data were assigned LOD and then used to calculate the disease burden.

## Data Availability

The data presented in this study are available upon request due to privacy restrictions.

## Supplementary Materials

The following supporting information can be downloaded at: https://www.mdpi.com/article/10.3390/nu16244381/s1, Table S1. The main modules of FDA-iRISK 4.2 and key input parameters. Table S2. Calculation of the LADD for Σ_5_AF from rice and its products-Iteration 1. Table S3. Calculation of the LADD for Σ_5_AF from rice and its products-Iteration 2.
