# Supplementary material for: Disease Burden Estimation of Hepatocellular Carcinoma Attributable to Dietary Aflatoxin Exposure in Sichuan Province, China
Source: Nutrients. 2024 Dec 19;16(24):4381. doi: 10.3390/nu16244381 (PMC11677878; doi:10.3390/nu16244381)
Supplement: Supplementary file 1 [file nutrients-16-04381-s001.zip › nutrients-3315066-supplementary.pdf]

Supplementary methods

S1. The main modules of FDA-iRISK 4.2 and key input parameters

FDA-iRISK 4.2 (FDA/CFSAN, College Park, Maryland, United States) is an online quantitative risk assessment tool released by the U.S. Food and Drug Administration (FDA). iRISK consists of four main modules: hazard, food, process, and risk scenario [1,2]. The dose-response model in the hazard module establishes the relationship between aflatoxin (AF) and hepatocellular carcinoma (HCC). The food module encompasses food types and consumption models, estimating the average daily amounts of AF-contaminated foods for different life stages. In the process module, interventions may impact final AF contamination levels. The risk scenario module combines these to simulate scenarios, estimating the lifetime average daily dose (LADD) of lifetime dietary AF exposure and the corresponding HCC disease burden. The risk scenario in this study was defined as "chronic exposure to a single hazard across multiple foods." The key input parameters are shown in Table S1.

Table S1. The main modules of FDA-iRISK 4.2 and key input parameters.

Module	Parameter	Input value
Hazard	Hazard Type	Chemical
	Hazard Name	Aflatoxins
	Dose Response Model	
	Exposure Type	Chronic
	Response Type	Linear by Slope Factor
	Slope [1]	0.000109 (Male 0.000105, Female 0.000113)
	Health Metric (DALY per case) [2]	12.37
Food	Food Type	Grain and its products, Nuts and seeds, et al.
	Consumption Model	
	Number of Consumers [3]	83,670,000
	Gender Ratio [3]	Male 50.54%，Female 49.46%
	Life Stages	0-6 months，7-12 months, 13-24 months, 2-6 years old, 7-17 years old, 18-65 years old and 66-average life expectancy [4]
	Average Daily Consumption	Linear Empirical Distribution (g/kg bw/day)
Process	Initial Unit Mass	1.0 kg (Fixed Value)
	Initial Prevalence [5]	100% (Fixed Value）
	Initial Contamination	See Table 1 in article (Fixed Value)
	Process Model	No Change
Risk Scenario	Single hazard, Multifood, Chronic exposure	

[1] The inverse of the AF intake amount corresponding to a 10% increase in lifetime HCC risk was estimated using the AFB_1 carcinogenic potency from JECFA, along with the average life expectancy from the Sichuan Population Health and Key Diseases Report of 2022.
[2] The DALY for each HCC case was calculated based on the data from the China Cancer Registry Annual Report of 2019.
[3] The number and gender ratio of consumers obtained from the Major Figures on 2020 Population Census of China.
[4] According to the Sichuan Population Health and Key Diseases Report of 2022, the average life expectancy is 77.91 years (75.26 years for males and 80.99 years for females).
[5] The prevalence represents the proportion of food units contaminated with AF.

S2. The calculation methodology and illustrative examples of the LADD

The FDA-iRISK model meticulously simulated diverse lifetime AF exposure patterns among consumers, generating tens of thousands of iterations to encompass the variability within the population. Each iteration depicted a unique exposure pattern, with some showing heightened exposure during childhood and youth, tapering off later in life, while others exhibited the opposite trend, with higher exposure in adulthood and old age.

The study's consumption data was structured as a cumulative empirical distribution of daily consumption rates, expressed in grams per kilogram of body weight. As different foods will be consumed by different fractions of the population in each life stage, the distribution describing the consumption level will necessarily include a proportion of consumers with zero consumption. For each iteration and life stage, FDA-iRISK randomly drew a single value from this distribution to simulate an individual's lifetime consumption pattern. The LADD was then calculated, representing the daily dose of AF ingested by the consumer, averaged across their entire lifespan and attributed to the specific food in question. The contribution of each life stage to the LADD was weighted according to its duration, thereby condensing the dynamic exposure profile into a single representative value ($LADD_R$). This LADD value was subsequently fed into a dose-response model to estimate the average risk of HCC per consumer.

Our research delved into simulating potential lifetime exposure patterns of AF at both mean and 95th percentile contamination levels across 20 food categories. Tables S2 and S3 showcase exemplary LADD calculations derived from iterations where randomly sampled consumption values mirrored high consumption patterns at either a young or older age among the whole population in Sichuan Province, China, respectively. For illustrative purposes, we assume a final mean AF concentration of 1.21 μg/kg in rice and its products, with a prevalence of 100%. These iterations exclusively represent two exposure scenarios involving AF in rice and its products. To obtain the LADD of Σ_5AF across all food categories, similar simulations would be conducted for the remaining food types.

Calculated the LADD of Σ_5AF intake across all studied food categories according to the following formulas:

$$LADD = \sum_{k=1}^{m} LADC_k \times C_k \times 100\% = \sum_{k=1}^{m} LADD_k \tag{S1}$$

$$LADC_k = \sum_{i=1}^{n} A_i \times Y_i \,/L \tag{S2}$$

Where $LADC_k$ is the lifetime average daily consumption of food category k (g/kg bw/day); $LADD_k$ is the LADD of Σ_5AF from food category k (ng/kg bw/day); C_k is the final AF content in food category k (μg/kg); *100%* is the final AF prevalence; *m* is the number of food categories; A_i is the daily consumption per kg bw for life stage i (g/kg bw/day); Y_i is the duration of life stage i (years); *n* is the number of life stages; and *L* denotes the average life expectancy.

Table S2. Calculation of the LADD for Σ_5AF from rice and its products-Iteration 1.

Parameters	Input values/Output values						
Life Stage	0-6 months	7-12 months	13-24 months	2-6 years old	7-17 years old	18-65 years old	65-77.91 years old
A_i, g/kg bw/day	0.00	0.00	0.00	8.22	9.05	5.14	4.61
Y_i, years	0.5	0.5	1	5	11	48	11.91
C_i, μg/kg	1.21						
P_i, %	100						
$LADC_i$	5.677 g/kg bw/day						
LADDi	6.87 ng/kg bw/day						

Table S3. Calculation of the LADD for Σ_5AF from rice and its products-Iteration 2.

Parameters	Input values/Output values						
Life Stage	0-6 months	7-12 months	13-24 months	2-6 years old	7-17 years old	18-65 years old	65-77.91 years old
A_i, g/kg bw/day	0.00	0.00	0.00	3.10	4.25	8.07	7.41
Y_i, years	0.5	0.5	1	5	11	48	11.91
C_i, μg/kg	1.21						
P_i, %	100						
$LADC_i$	6.904 g/kg bw/day						
LADDi	8.35 ng/kg bw/day						

Reference

1. Chen, Y.; Dennis, S.B.; Hartnett, E.; Paoli, G.; Pouillot, R.; Ruthman, T.; Wilson, M. FDA-iRISK—A Comparative Risk Assessment System for Evaluating and Ranking Food-Hazard Pairs: Case Studies on Microbial Hazards. *J. Food Prot.* **2013**, *76*, 376–385.
2. Food and Drug Administration Center for Food Safety and Applied Nutrition; Joint Institute for Food Safety and Applied Nutrition; Risk Sciences International. FDA-iRISK® version 4.2. FDA CFSAN. College Park, Maryland. Available at https://irisk.foodrisk.org/.
